# Voice Outcomes in Laryngotracheal Stenosis: Impact of the Montgomery T-tube

**Published:** 2018-01-10

**Authors:** Vaninder K Dhillon, Lee M Akst, Simon R Best, Alexander T Hillel

**Affiliations:** Department of Otolaryngology, Johns Hopkins University, USA

**Keywords:** Montgomery T-tube, Laryngotracheal stenosis, Voice, Quality of life, Endoscopic techniques

## Abstract

**Objectives:**

Montgomery T-tubes enable patients with laryngotracheal stenosis to maintain airway patency. They also restore the ability to phonate in many patients. The primary objective is to compare voice quality of life outcomes in patients before and after Montgomery T-tube placement. The secondary objective is evaluating complications associated with T-tube placement.

**Methods:**

Retrospective chart review of patients with T-tubes for laryngotracheal stenosis from 2012–2016. Patient demographics, Voice-Related Quality of Life (VRQoL) scores, indication for t-tube placement, t-tube duration and complications were analyzed.

**Results:**

Thirteen patients were included. The most common indication for T- tube placement was grade III–IV stenosis with aphonia/significant dysphonia (n=7, 54%). Other indications were grade III/IV stenosis who desired T-tube over tracheostomy (n=2, 15%), primary glottic stenosis (n=3, 23%), and primary tracheomalacia (n=1, 8%). There was a statistically significant improvement (p<0.05) in VRQoL after T-tube placement. Five patients (38%) went from aphonia to voicing. Granulation tissue was the most common complication related to T tube placement. There were no deaths related to T-tube placement after two years.

**Conclusion:**

Montgomery T-tubes can restore phonation in a population of patients with iatrogenic high grade stenosis who are aphonic/severely dysphonic with traditional tracheotomies. The complication rate must be considered, with granulation tissue formation the most common.

## Introduction

Montgomery T-tubes were first described by William Montgomery in 1965 [1,2]. The original T-tubes were combined stents and tracheostomy tubes for patients with subglottic or upper cervical stenosis. They were designed in the shape of a ‘T’ with a proximal limb which bypassed the stenosis, a distal limb down to carina, and an external limb that communicated through the cutaneous stoma, like a tracheostomy tube [2]. Original Montgomery tubes were designed as temporary stents that would be placed 1) as a bridge to definitive reconstructive surgery, 2) as an adjunct to primary surgical intervention, 3) as a therapeutic intervention in the post-operative period with partial dehiscence or 4) in patients with recurrence of disease [3,4]. As a stent placed within the segment of stenosis, the T-tube was believed to remodel the airway so that it could eventually be removed with improved airway patency. The ultimate goal was to decannulate a patient from a T-tube as a definitive treatment for laryngotracheal stenosis. Montgomery described the original placement of the T-tube through an anterior laryngofissure, an open incision through the thyroid cartilage, which was then fastened to the laryngeal skeleton until removal either weeks or months later depending on timing of reconstruction [1]. The advancement of open techniques to endoscopic ones has increased the prevalence of T-tubes across specialties. Endoscopic techniques for placement through the use of laryngoscopy and bronchoscopy have been described widely within the thoracic literature [3,5–7]. The prevalence of T-tubes within otolaryngology is relatively small, less common than the prevalence of tracheostomy for airway management and the risk of complications like granulation tissue, obstruction such as mucous plugs, and glottic scarring [3]. There is little published about the impact of T-tube placement for phonation. Furthermore the indications for placement to improve voice and the use of T-tubes in long term airway management have not been well studied. While there may be a hesitance to use T-tubes secondary to the risk of complications, the role they have in airway management and voice restoration for a certain demographic of patients may, in fact, prove more beneficial than tracheostomy. In this study, we evaluate voice in laryngotracheal stenosis patients with Montgomery T-tubes. We look at demographic factors, the etiology of stenosis and indications for placement. Our primary outcome is to measure voice outcomes using VRQoL scores in patients before and after Montgomery T-tube placement in order to describe the improvement in phonation for these patients. The secondary outcome is complications of T-tube placement, and whether the complication requires replacement with a tracheostomy. Our hypothesis is that Montgomery T-tubes can provide a long-term alternative to tracheostomy, and serve to maintain airway while optimizing voice for the patient.

## Materials and Methods

### Study design and data collection

This study was conducted under the approval of the Johns Hopkins Institutional Review Board; IRB number #NA81469. A retrospective chart review was performed for all patients seen by two fellowship trained laryngologists at Johns Hopkins University from the years 2012 to 2016 who had placement of a Montgomery T-tube. Demographic data was collected for all patients. Inclusion criteria included patients with T-tubes placed at Johns Hopkins University. Exclusion criteria included patients with cancer, or active malignant disease contributing to laryngotracheal stenosis. Etiology of laryngotracheal stenosis was divided into iatrogenic causes (due to intubation and/or tracheostomy) and ‘Other’ category that included recurrent respiratory papillomatosis, supracricoid laryngectomy with tracheal stenosis, primary tracheomalacia and penetrating trauma to the larynx. Indications for T-tube placement were recorded. The indications for T-tube placement included grade II/IV stenosis who desired T tube over tracheostomy, primary glottic stenosis and primary tracheomalacia. All patients in this study had pre-existing tracheostomy stomas. Outcomes measured were voice related quality of life scores (VRQoL) and complications from placement of T-tube. VRQoL were collected at each of these visits both before and after T-tube placement, for each patient. Calculated scores were then recorded [8]. The voice related quality of life (VRQoL) was established as a subjective measure to qualify phonation [8,9]. It has been validated as a 10-item survey that reliably evaluates the change in perceptual voice quality ratings before and after treatment for dysphonic patients [10,11]. Operative reports were reviewed with regards to Montgomery T-tube sizes and techniques for placement. Surgical technique was collectively obtained for both laryngologists. Complications were defined as granulation tissue, difficulty ventilating, dyspnea, need to resize T-tube, tracheitis and migration of T-tube. The need for tracheostomy replacement was determined for each complication. The length of time each patient maintained a T-tube was also recorded. Length of time was recorded from initial placement of T-tube to date last recorded with T-tube.

### Surgical technique

A Universal modular glottis cope or dedo laryngoscope was placed trans-orally for visualization of the proximal limb of the T-tube. A hemostat was used to place the proximal limb with hemostat through tracheostomy stoma, unfurling it by using laryngeal forceps through the laryngoscope from above. The proximal limb was positioned below the level of the vocal cords using multiple techniques. One surgeon (A.H.) used umbilical tape in seven patients to assist proximal placement of the T-tube. The distal limb was placed using the hemostat through the tracheostomy stoma. One surgeon (S.B) used balloon dilation on two patients to assist in opening the distal limb through the laryngoscope so that the silicone walls opposed the tracheal lumen. A Hopkins rigid 0 degree telescope was used by both surgeons for final confirmation of patency within the laryngotracheal lumen visualization distal to carina. Patient follow up included serial examinations in clinic every 2–3 months for evaluation and debridement of t-tube secretions, if necessary. All patients kept their T-tubes capped, except to suction. T-tube exchange was done on average every 4–6 months over the course of a four-year follow up.

### Data analysis

Categorical data were compared using Fisher exact tests for variables with less than 10 counts (Table 1). We compared interval data between 2 groups using the Student t-test for variables with a parametric distribution (Table 2). Statistical significance was p<0.05.

## Results

Demographics of the 13 patients included in this study are shown in Table 1, stratified by etiology. The average age was 45.8 years. 100% of our patients had pre-existing tracheostomy stomas. The most common indication for T- tube placement was grade III–IV stenosis with aphonia/significant dysphonia (n=7, 54%). Other indications were grade III/IV stenosis who desired T-tube over tracheostomy as a closed airway system (n=2, 15%), primary glottic stenosis (n=2, 15%), grade I–II stenosis with associated glottic stenosis (n=1, 8%), and primary tracheomalacia (n=1, 8%). The average length of time for patients with iatrogenic causes was 2.44 years and for the other category was 1.19 years. Overall, six patients continue to have a Montgomery T-tube, with an average duration of 3.3 years (range: 0.33 year to 8.42 years). The etiology of laryngotracheal stenosis was divided into iatrogenic causes (n=9, 69%) and other (n=4, 31%). Iatrogenic causes were those due to intubation and/or tracheostomy. Five out of thirteen (38%) patients were morbidly obese with a BMI>30, and all five patients had iatrogenic etiology for their stenosis (p>0.05). All five patients who had a history of open reconstructive procedures were also within the iatrogenic group (p>0.05). The four patients in the other category included recurrent respiratory papillomatosis, supracricoid laryngectomy with tracheal stenosis, primary tracheomalacia and penetrating trauma to the larynx. Table 2 shows the individual VRQoL score for all thirteen patients before and after T tube placement. The average calculated VRQoL score between initial scores and those post-operatively was 43.5 to 79 (p<0.05), with improvement in the post T-tube score compared to the pre-placement score. Two patients had a worse calculated VRQoL following T-tube placement. There were a total of 12 complications amongst 13 patients in our study (Table 3). There was no need to replace a T-tube with a tracheostomy tube for granulation tissue in comparison to the other complications. Six of thirteen (46%) patients continue to have T-tubes. Of the seven remaining patients, three had replacement of their T-tube for a tracheostomy—two of them at outside institutions for ventilatory needs, and one for obstructive secretions from the T-tube. Two patients (15%), one with iatrogenic high-grade stenosis, and one with recurrent respiratory papillomatosis, were transitioned back to tracheostomy and then decannulate successfully. Two patients died of unrelated causes.

## Discussion

Our study is one of the few studies to demonstrate how Montgomery T-tubes can provide improvement in phonation [5,12]. There was a statistically (p<0.05) and clinically significant improvement in VRQoL scores after T-tube was placed. Eleven of 13 patients had improvement in VRQoL with an average improvement of 35.5. The voice outcomes highlight the benefit of T-tubes in restoring complex airway patient’s ability to voice or significantly improve from baseline dysphonia. Furthermore, the long-term utility of T-tubes in certain patients is demonstrated as feasible with appropriate follow-up, in addition to its use as a temporary airway stent as described in the literature [3,5,12]. While we had a relatively high complication rate, the majority of our complications were treatable, and none resulted in patient intolerance to T-tubes. The complication rates vary across published studies Shi et al. [3–5,13]. Described complication rates in 546 patients, among them hemoptysis (1.5%), postoperative infection (1.1%), wound dehiscence (0.5%), laryngeal obstruction (2.4%), aspiration (2.2%), and postoperative tracheoesophageal fistula (0.4%) an older study done by Wain et al. [3]. showed a complication rate of 20% secondary to specifically obstruction from the T-tube. The most common complication for patients with T-tubes was granulation tissue, consistent with other publications [3,10,12]. Interestingly, in our study, granulation tissue did not lead to replacement with a tracheostomy tube, perhaps due to regular follow-up allowing for rapid identification and medical treatment. The other complications, including dyspnea, difficulty ventilating, tracheitis and T-tube migration necessitated exchange back to a tracheostomy. Furthermore, we did not have a complication related to plugging of the T-tube postoperatively. One explanation for our high complication rate may be unique to our patient cohort that is biased by its small size. It may also be that we were comprehensive in our definition of complication. There is variability in the literature in terms of what is included and defined as a complication. Some studies only describe obstruction related to the T-tube as the complication [10], while others highlight a few common complications [12]. Our study’s relatively high rate of complications does emphasize the risks related to T-tube placement, need for regular follow-up, and consideration of appropriate candidates. Improved phonation and comfort with a T-tube (versus a tracheostomy) must be balanced in these patients with the likelihood of complications. Physicians must be comfortable that the patients can responsibly take care of their t-tube and are capable of managing in an emergency due to airway obstruction from plugging [14]. While the purpose of this study is not to characterize the indications for T-tube placement, there are patterns that emerge from our data. Candidates included patients with high-grade stenosis that failed or were not candidates for cricotracheal or tracheal resections, or those with tracheomalacia. Nine patients had iatrogenic causes for their laryngotracheal stenosis, seven of which were high grade (Grade IIIIV). Shi et al. [4] showed that the rate of decannulation from a T-tube gradually declined as Cotton Myer grades for stenosis increased. All five obese patients were in the high-grade stenosis category, many of which were not candidates for cricotracheal or tracheal resection. While obesity has not been associated as a risk factor for failure of open airway reconstruction, some surgeons have strict criteria that exclude these patients from undergoing open resection [14]. We had one patient with primary tracheomalacia, which is another indication for permanent T-tube stenting of collapsing tracheal walls [12,16]. Beyond our indications already described for T-tube placement, it is also important to note that patients who are unable to use a Passy Muir valve and/or have subjective dysphonia with a tracheostomy tube should be considered for a T-tube. T-tubes may provide structure for the tracheal wall allowing for sufficient airflow for phonation that would otherwise not suffice with a traditional tracheostomy tube. Finally, patient preference for an alternative closed airway system, comfort and alternative to tracheostomy maintenance are also soft reasons for T tube placement. Furthermore, this study demonstrated the role of the T-tube for long-term airway management in addition to its role as a short-term stent. Overall 6/13 patients maintained a T-tube >2 years. Two patients who had the T-tube less than two years in duration were successfully decannulate after a T-tube stent trial. Six patients maintained a T-tube, with the average duration of placement 3.3 years. When stratified according to etiology of laryngotracheal stenosis, the average length of a T-tube in patients with iatrogenic causes was 2.44 years as compared to 1.19 years in the other category. There are limitations in this study. It is a small retrospective case series. However it is the first to focus on the otolaryngology outcome of voice. While this represents one of the larger Otolaryngology T-tube case series, the small yet disparate LTS cohort of patients who receive T-tubes demonstrates the difficulty in a single institutional study. A larger multi-institutional prospective study with a longer follow up could prove beneficial. Furthermore our cohort may be biased based upon our patient demographic and etiology of laryngotracheal stenosis seen at our tertiary center. Our voice outcome measures focused around a single quality of life metric. Future studies looking at voice outcomes in patients with Montgomery T-tube would benefit from other quality of life metrics, as well as objective measures such as acoustic analysis and perceptual evaluation of voice recordings.

## Conclusion

To our knowledge, our study is the first to describe the role of a Montgomery T-tubes in providing a long-term solution for voice in individuals with laryngotracheal stenosis. A majority of our cohort demonstrated clinically significant improvement in voice related QOL scores. Long-term use of a T-tube was feasible to provide voice and airway. The complications associated with T-tubes are not insignificant, and thus the risk for T-tube placement should be balanced with the benefits in voice and breathing on an individual patient basis. Overall our study identifies the benefit of Montgomery T-tubes in obtaining optimal voice outcomes, an important factor to consider within an otolaryngology and airway practice.

## Figures and Tables

**Table 1:** Demographic and baseline characteristics of the study subjects.

N=13	Overall	Etiology of laryngotracheal stenosis^1^	
Age in years M	45.8 (range: 26–68)	Iatrogenic (n=9)	Other (n=4)
Gender N (%)			
Female	10 (77%)	7 (78%)	3 (75%)
Male	3 (23%)	2 (22%)	1 (25%)
History of tracheostomy N (%)	13 (100%)	9 (100%)	4 (100%)
Cotton-Myer Classification^4^	I–II	1 (12%)	0 (0%)
	III–IV	7 (88%)	2 (100%)
Obesity^2^	5 (100%)	5 (100%)	0
History of open procedure^3^ N	5 (100%)	5 (100%)	0
Duration of T-tube(average years) N		2.44	1.19

1 **Iatrogenic:** Intubation trauma and subsequent tracheostomy placement

**Other:** Recurrent respiratory papillomatosis, supracricoid laryngectomy with tracheal stenosis, primary tracheomalacia and penetrating trauma to the larynx

2 **Obesity:** BMI >30

3 **Open procedure:** tracheal resection, any open reconstruction of the trachea

4 **Cotton-Myer Classification** I–II: <70% stenosis; III–IV: 71% to 100% stenosis

**Table 2:** Voice Related Quality of Life (VRQoL) measure pre and post T tube placement.

Patient	Etiology of Laryngotracheal Stenosis	VRQoL pre T tube	VRQol post T tube	Aphonic Prior to T tube placement
1	Iatrogenic	42.5	92.5	No
2	Iatrogenic	0	100	Yes
3	Iatrogenic	0	0	No
4	Iatrogenic	0	100	Yes
5	Other	70	72.5	No
6	Other	75	20	No
7	Iatrogenic	0	100	Yes
8	Iatrogenic	92.5	97.5	Yes
9	Iatrogenic	80	100	No
10	Iatrogenic	95	100	No
11	Other	60	65	No
12	Iatrogenic	30	90	Yes
13	Other	70	90	No
Average VRQoL (N=13)		43.5^1^	79	5 (Total N)

1 P<0.05

**Table 3:** Complications after Montgomery T tube placement.

Complications	N	Requiring tracheostomy replacement
Granulation tissue	6	
Difficulty ventilating (OSH)	1	1
Dyspnea/re-size T tube	2	1
Tracheitis	2	1
Migration of T tube	1	1
